# Patterns of Early-Life Gut Microbial Colonization during Human Immune Development: An Ecological Perspective

**DOI:** 10.3389/fimmu.2017.00788

**Published:** 2017-07-10

**Authors:** Isabelle Laforest-Lapointe, Marie-Claire Arrieta

**Affiliations:** ^1^Department of Physiology and Pharmacology, University of Calgary, Calgary, AB, Canada; ^2^Department of Pediatrics, University of Calgary, Calgary, AB, Canada

**Keywords:** microbiome, early-life events, immune development, microbial ecology, diversity, keystone taxa

## Abstract

Alterations in gut microbial colonization during early life have been reported in infants that later developed asthma, allergies, type 1 diabetes, as well as in inflammatory bowel disease patients, previous to disease flares. Mechanistic studies in animal models have established that microbial alterations influence disease pathogenesis *via* changes in immune system maturation. Strong evidence points to the presence of a window of opportunity in early life, during which changes in gut microbial colonization can result in immune dysregulation that predisposes susceptible hosts to disease. Although the ecological patterns of microbial succession in the first year of life have been partly defined in specific human cohorts, the taxonomic and functional features, and diversity thresholds that characterize these microbial alterations are, for the most part, unknown. In this review, we summarize the most important links between the temporal mosaics of gut microbial colonization and the age-dependent immune functions that rely on them. We also highlight the importance of applying ecology theory to design studies that explore the interactions between this complex ecosystem and the host immune system. Focusing research efforts on understanding the importance of temporally structured patterns of diversity, keystone groups, and inter-kingdom microbial interactions for ecosystem functions has great potential to enable the development of biologically sound interventions aimed at maintaining and/or improving immune system development and preventing disease.

## Introduction

Recent advances in immune-mediated disease research have provided a considerable body of proof revealing the importance of the early gut microbiome for neonatal immune system development and disease pathogenesis [see Ref. (1) for a review]. The drastic increase of allergies and other immune-mediated diseases in industrialized countries has been hypothesized to be a result of deficiencies in the exposure to microbial organisms and their products, resulting in impaired immune system development, a concept first introduced as the hygiene hypothesis (2, 3). Pioneer work has identified the first 6 months after birth as a “window of opportunity” (4–7) during which contact with specific microbe-associated molecular patterns (MAMPs) triggers a cascade of reactions crucial for infant gut maturation (8–10). Disrupting early gut community succession may lead to dysbiosis, a state of ecological imbalance ensuing when the community loses key taxa, diversity, and/or metabolic capacity. This state can lead to a reduction of colonization resistance, allowing for a subsequent bloom in opportunistic pathogens [(11); for a definition of relevant ecological concepts refer to Table 1]. Concomitantly, microbial dysbiosis during infancy may also lead to health-related consequences in the neonatal stage or later in life. Preterm neonates can develop necrotizing colitis (NEC), a life-threatening disease strongly associated with microbial dysbiosis (12). Infants may also experience an elevated risk of developing inflammatory diseases such as asthma and allergies (13, 14), type 1 diabetes (15, 16), celiac disease (17), inflammatory bowel disease (18, 19), and obesity (20, 21) when exposed to a microbial dysbiosis early in life. Thus, studying the patterns of microbiome assembly and how disturbances to this process reflect in the developing immune system is of utmost importance to understand the origin of human diseases responsible for enormous health and economic burden to societies.

**Table 1:** Definition of selected ecological concepts.

Concept	Definition
Complex adaptive system	A system composed of a multitude of autonomous and interdependent actors that share a variety of interactions, and behave as a unified whole in reacting and adjusting to changes in the environment (22)
Emergent property	A system’s property that its components lack individually
Stochasticity	The unpredictable fluctuation of environmental conditions
Temporally structured ecosystem	An ecosystem in which emergent properties (e.g., taxonomic and functional diversity, resilience) rely on a conserved succession of events ordered in time
Richness	The number of “species” in a community
Alpha-diversity	The number of “species” and their abundance within a community or the mean in a collection of communities (i.e., Shannon index)
Beta-diversity	The absolute turnover in community composition often measured as communities’ pair-wise dissimilarity in microbial ecology, also defined as the ratio between regional and local species diversity
Taxonomic diversity	The number and the relative abundance of species or taxa in a community
Functional diversity	The variety of processes or functions in a community that are important to its structure and dynamic stability
Resilience	A system’s or community’s capacity to promptly return to its initial state after a perturbation
Resistance	A system’s or community’s capacity to resist or impede changes in its state while withholding a perturbation
Selection	A key evolutive process in which genetic and environmental pressures determine which organisms succeed at survival and reproduction
Keystone species	An exceptionally important species whose presence is crucial in maintaining the organization and diversity of the ecological community (23, 24)
Succession	A pattern of changes in specific composition of a community after a radical disturbance or after the opening of a new patch in the physical environment for colonization (25)

The infant gut microbiome is a complex ecosystem involving a great number and diversity of members (e.g. bacteria, phages, fungi, viruses, protozoans) that interact in a spatially and temporally structured environment (26–28). The neonatal gut microbiota can be considered a complex adaptive system in which both low-level local interactions and selection mechanisms combine to create high-level patterns (22). Complex adaptive systems are non-linear (output not proportional to the input, thus impeding predictability) in that they are heavily influenced by stochastic temporal events that result in a plethora of variable outcomes (22). The infant gut microbiome supports a set of emergent properties contributing to host physiology, including nervous, metabolic, and immune development (29–31), as well as tissue differentiation (32, 33). The emergent properties of a complex adaptive system are considered to be supported by combinations of taxonomic and/or functional diversity, as well as key taxonomic and/or functional groups, both of which insure community resilience (22), and increase the difficulty of attributing a cause–effect relationship to unique features or groups. Therefore, including community ecology theory to study the temporal dynamics of the infant gut microbiome has the potential to provide key information about its influence on the host immune system maturation.

Until 2 years of age, the human infant microbiome remains highly heterogeneous and lacks stability (34), being influenced by temporally structured environmental factors such as (1) maternal factors (35–37), (2) birth (38–41), (3) neonatal nutrition (27, 42, 43), and (4) other non-temporally structured factors, such as antibiotic treatments (41, 44, 45). The initial intestinal bacterial community composition of vaginally born infants involves higher levels of a multitude of bacterial groups (e.g., *Atopobium, Bacteroides, Clostridium, Escherichia coli, Streptococcus* spp. and *Prevotella*), while the community of infants born by C-section is dominated by skin-related taxa including *Staphylococcus* spp. (38). Key bacterial groups are also transferred to the infant by breastfeeding: *Bifidobacterium* and *Lactobacillus* (46–48). The multiple studies that have shown how intestinal dysbiosis can lead to detrimental immune-mediated outcomes (e.g., asthma, allergies, NEC, etc.) [see Ref. (30) for a review] suggest that the human immune system relies on an evolutionary conserved temporally structured succession of microbiome assembly. Unraveling the links between the temporal mosaics of the gut microbiome (structured succession patterns) with the emergent properties of this ecosystem (e.g., taxonomic and functional diversity, resilience, etc.) is key to improve our understanding of the importance of the infant microbiome for the development of the immune system.

The successful identification of the mechanisms linking the infant gut microbiome and immune development depends on our capacity to disentangle the relative effects of multiple factors (host genetics, environmental factors), key actors (e.g., *Bacteroidetes, Bifidobacterium*, etc.) and their interactions. Resilience, the ability of a system to adjust its activity to retain its basic functionality after a disturbance, is a crucial property of complex adaptive systems (49) and could be a key characteristic protecting the infant gut microbiome from reaching a dysbiotic state. Here, we review the recent findings on the links between infant gut microbiota and immune system maturation. Our review highlights the reliance of the neonate immune system development on a complex set of host-specific, environmental, temporal, and self-organizing characteristics of the infant gut microbiome. We propose that future studies should consider multi-level dynamics of the infant gut microbial community by disentangling the ecosystem reliance on (1) temporally structured patterns of alpha- and beta-diversity, both taxonomic and functional; (2) keystone species or microbial groups; and (3) inter-kingdom interactions. This will require a conceptual framework based on the understanding that the infant gut harbors a complex and diverse set of microbial species interacting in a temporally structured, multi-level, and non-linear network. Rightfully recognizing these structural characteristics has the potential to enable the identification of disturbance thresholds threatening the healthy development of the infant gut microbiome and its role in immune system training.

## Age-Dependent Immune System Development

Multiple studies and comprehensive reviews discuss how the maturation of the immune system relies on the exposure to MAMPs (50–52). Here, we discuss the recent findings demonstrating that the efficiency of microbial exposure in immune system training can be age dependent, suggesting the importance of microbial composition and infant gut microbiome temporal succession patterns.

The gastrointestinal tract is already anatomically and functionally developed at birth in full-term infants, yet important aspects of its maturation occur postnatally and depend on exogenous stimulations with microbial cells, metabolites, hormones, growth factors, and antigens (53, 54). Recent studies in murine models have revealed that several aspects of immune development are more permissive to microbial-mediated changes during early life, and that certain microbial taxa are crucial in these interactions. For instance, oral administration of *Bifidobacterium breve* was effective in inducing proliferation of FoxP3-positive regulatory T cells (FoxP3^+^ Tregs) only if administered during the pre-weaning stage in mice (55). This age-dependent promotion of an important tolerogenic immune cell was also shown to be species specific, thereby suggesting that the tolerogenic gut immune response may have adapted to respond to specific—and important—bacterial taxa. *Bifidobacterium* species and subspecies are dominant members of the infant gut microbiome (56) and are strong modulators of the immune response (57). Their role as keystone taxa of the infant gut is proposed later in this review. Another microbial species that cause an age-dependent immune effect is the *Helicobacter pylori*, which ameliorated airway hyperresponsiveness more effectively when administered before weaning in two relevant mouse models (58), although it remains unclear if and when this bacterium colonizes the infant gastrointestinal tract.

While age-dependent modulation of the host’s immune response can be attributed to specific microbial taxa, most studies point to global changes in the microbial community (diversity shifts, and metabolites of poly-microbial origin) as drivers of immune development. Cahenzli et al. (59) showed that regulation of IgE responses and amelioration of antigen-induced oral anaphylaxis is dependent upon increased microbial diversity during early life. Their work thus suggests that there may be a diversity threshold necessary for proper maturation of these Th2 immune mechanisms. Furthermore, several other studies have demonstrated the immune consequences of the disruption of the early-life gut microbial community using antibiotics. Antibiotics induce drastic compositional and diversity shifts that lead to changes in crucial immune functions, including Treg proliferation (60, 61), IgE response (60, 62), Th-17 response (61, 63), and basophil-mediated Th2-cell responses (62). Given the influence exerted by these immune functions on widespread tissues and systems, it is not surprising that antibiotic-induced immune alterations during early life in animal models aggravate autoimmune diabetes (61, 64), allergic lung inflammation (60, 62, 63), inflammatory chronic colitis (65), and obesity (20, 21).

Early-life immune development is also reliant on the actions of a group of bacterial metabolites known as short-chain fatty acids (SCFAs). These compounds are direct by-products of bacterial colonic fermentation and are produced at very high rates (66). Acetate, propionate, and butyrate are the SCFAs produced in highest concentrations in the human gut, and are rapidly taken up by the gut epithelium through passive and active transport mechanisms (67). SCFAs are essential energy sources for colonocytes cells in the mammalian gut, and are precursors for gluconeogenesis, liponeogenesis, and protein and cholesterol synthesis (68). Among many of their immune functions [reviewed in Ref. (66)], SCFAs have been shown to induce extrathymic proliferation of Foxp3^+^ T cells (68–70), which orchestrate peripheral tolerance in mucosal tissues. This critical immune function of SCFA has been shown to be relevant for the offspring even if exposure occurred before birth. Oral administration of acetate during pregnancy was sufficient for the priming of FoxP3^+^ Treg cells and preventing allergic airway inflammation in the adult offspring (36), suggesting that *in utero* exposure to maternal gut microbial metabolites contributes to the development of immune functions in the airways of the offspring.

In addition to interactions with the developing immune system, a recent study by Kim et al. (71) suggests that the early gut microbiome confers colonization resistance through the production of bacterial metabolites resulting from age-dependent colonization with key bacterial taxa. Clostridial species from *Clostridium* clusters IV and XIVa, which increase in abundance with age, induced colonization resistance to intestinal mouse pathogens *Salmonella enterica* subsp. *typhimurium* and *Citrobacter rodentium*. Interestingly, the conferred mechanism of resistance is unrelated to immune adaptors MyD88 and TRIF, and independent of B and T cell function. The settlement of *Clostridia* in the gut of GF mice was also greatly reduced by the absence of neonatal bacteria, which may help explain the increased susceptibility of newborns and young infants to these GI infections.

Collectively, these studies constitute compelling evidence that key taxa, microbial community diversity, and bacterial metabolites constitute modulatory triggers of host immune function maturation. Although considerable research effort has been made, a great deal of the age-dependent processes through which microbial exposure drives immune system development remains to be identified. The infant gut microbiome temporal succession patterns, driven by birth, weaning, and introduction of solid foods, match marked changes in host immune function (72, 73). Therefore, future studies designed during these events, such as human longitudinal cohorts, hold great potential to improve our understanding of the dynamics at play.

## Temporally Structured Environmental Factors

**Figure 1:**
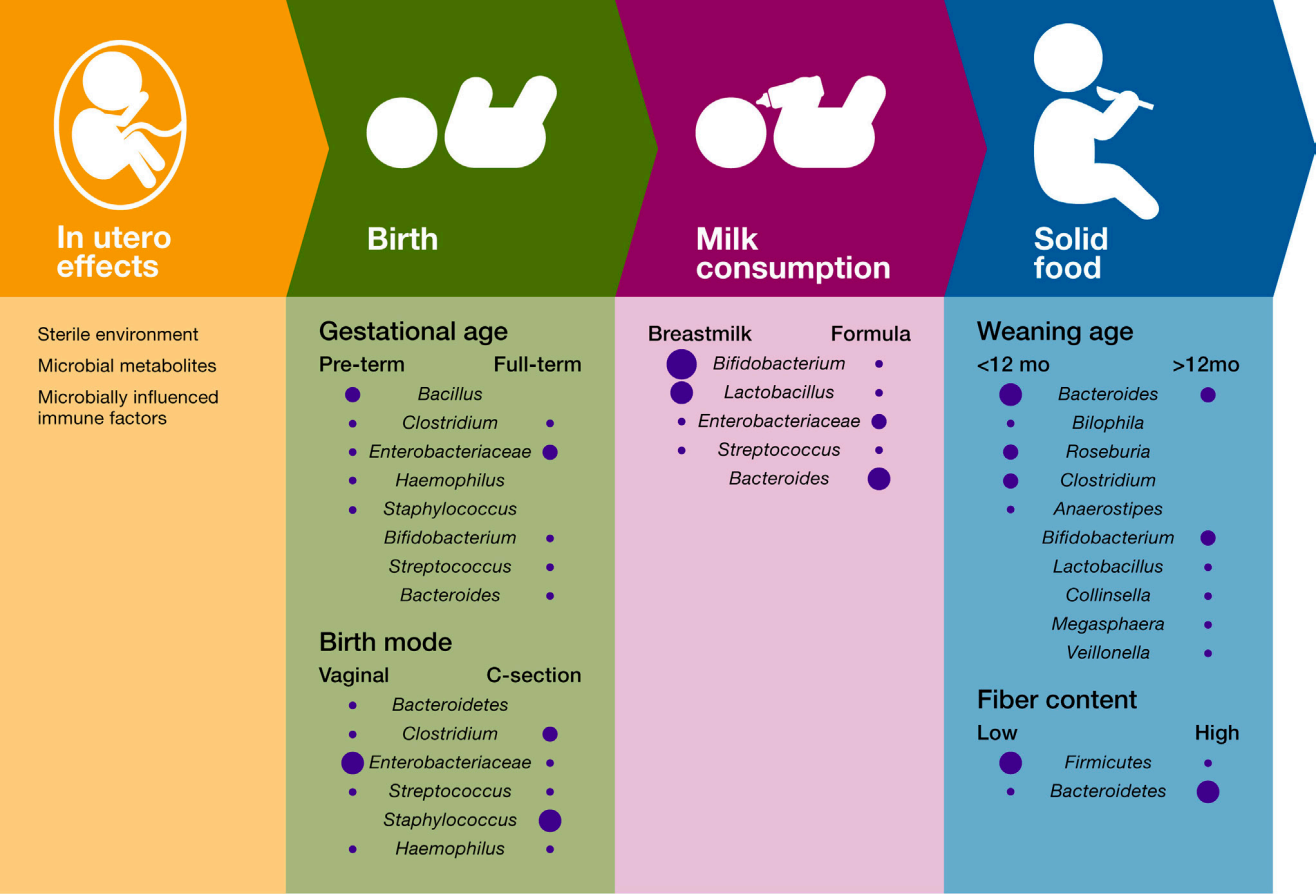
Influence of temporal succession events and environmental factors on the infant gut bacterial microbiome. Only the most important differences in bacterial composition are included for each variable, and the size of the circle is proportional to the relative abundance of the bacterial taxa.

Succession in ecology is defined as the pattern of changes in a community after a disturbance or after the opening of a new patch to colonization (74). Correspondingly, succession in the infant gut microbiome starts with the arrival of pioneer species that transform the gut habitat and enable the settlement of first succession species. The identity of the infant gut pioneer and first succession species is influenced by factors such as maternal factors (e.g., body weight and stress) (35–37), delivery mode (38–41), and type of milk consumption [(27, 42); Figure 1]. The temporal structure of these environmental factors contributes to the identity and dynamics of the infant gut microbiome and plays a role in the immune system training.

### Prenatal Life

Even before birth, fetal immune development relies on microbial products present in the placenta. In an experimental system in which germ-free mice were transiently colonized with genetically engineered *E. coli* HA107, maternal gut colonization influenced the offspring’s immune system by increasing the intestinal group 3 innate lymphoid cells and F4/80^+^CD11c^+^ mononuclear cells (iMNCs), and strongly altering the offspring’s intestinal transcriptional profiles (37). These early shifts in the offspring immune system improved the capacity of the pups to avoid inflammatory responses to MAMPs and intestinal microbes’ penetration, thus suggesting that microbial training of the immune system starts *in utero* (37). Despite some reports suggesting that fetal colonization may begin *in utero* (75, 76), lack of appropriate contamination controls and failure to show bacterial viability in these studies yields this work inconclusive and inadequate to disproof the currently accepted view of the placenta as a sterile environment (77). More importantly, several studies have shown that early colonization of the infant gut is strongly driven by mode of birth (39–41, 78), thus suggesting that direct colonization of the infant gut most likely begins after membrane rupture, during labor and birth. For example, Backhed et al. (39, 40) showed that the gut microbiome of vaginally born infants exhibited an enrichment in *Bifidobacterium, Bacteroides, Escherichia*, and *Parabacteroides*. In comparison, the gut microbiome of infants born through cesarean sections (C-sections) was enriched in microbes associated with the skin, the mouth, and the surrounding environment.

### Birth

The infant’s gut habitat changes rapidly after birth with facultative anaerobes species (e.g., *E. coli, Staphylococcus*, and *Streptococcus*) colonizing first and consuming the available oxygen (79). A longitudinal study following 39 infants from birth demonstrated that mode of delivery impacts *Bacteroides* populations in the infant’s gut microbiota between 6 and 18 months of age (41). Yassour et al. (41) showed that, in comparison with vaginal-birth, most infants born by C-section lacked the presence of the *Bacteroides* genus until about 6–18 months of age. Their work also showed that a higher abundance of *Bifidobacterium* species, both in C-section and vaginally delivered infants, was detected concomitantly with a lower abundance of *Bacteroides*, suggesting that infant gut microbial communities are also influenced by microbe–microbe interactions. Delayed colonization with *Bacteroides* species was also associated with cesarean sections in a study of 24 infants (80), a finding that was linked to lower levels of Th-1-associated chemokines CXCL10 and CXCL11 in blood. *Bacteroides* species are important and extremely common members of the human gut microbiome, capable of fermenting a variety of fibers in the colon (81) and modulating the immune system (their potential as a keystone taxa and role in immunomodulation is discussed later in this review). Hence, vertical transmission during vaginal birth is likely a structured environmental factor that promotes colonization by members of this influential bacterial group.

Gut microbiome differences driven by mode of birth have been reported in almost all microbiome infant studies that recorded this variable (28, 38, 82–87). Although the cumulated evidence points to the mode of birth being a major influence in the gut pioneer microbiome, one recent study performed on 115 infants showed no differences in the meconium microbial communities between both mode of birth (C-section and vaginal delivery) (88). Unfortunately, key bio-statistical parameters of their analyses are missing from the paper, crucial information to assess the robustness of their results. What still remains unclear is how long these differences last, with only a few reports showing differences beyond early childhood (82). Nonetheless, changes in important microbial groups, community diversity, or functions during this critical and permissive window of immune development are likely to induce immune alterations that may remain beyond the age at which these taxonomic differences are no longer detectable.

Intriguingly, the taxonomic identity of pioneer colonizers not only depends on birth but also on gestational age. A 1-month longitudinal study of 58 preterm infants in a neonatal intensive care unit showed that time post-conception can also impact the type of early colonizers in the premature gut yet not the pattern of bacterial succession (89). Members of the *Bacilli* class appear as the initial colonizers in premature infants, which contrasts with the initial colonization with *Enterobacteriaceae* members in most term babies (Figure 1). In addition, this study showed that gut microbiome follows a progression strongly determined by host biology factors, suggesting that, during the first month after delivery, the genetic and physiologic characteristics of the preterm infant gut drive a conserved pattern of succession in gut microbiome.

### Milk Consumption

In addition to mode of birth and post-conceptional age, diet during early infancy strongly impacts community structure and diversity. Comparisons between breastfed and formula-fed infants have shown that *Bifidobacterium* spp. and *Lactobacillus* spp. predominate in breastfed infants whereas formula-fed infants exhibit higher proportions of *Bacteroides* spp., *Clostridium* spp., *Streptococcus* spp., *Enterobacter* spp., *Citrobacter* spp., and *Veillonella* spp. (39, 40, 90–93). Breast milk can modulate the infant gut microbiome through different mechanisms. First, human breast milk contains a significant number of bacteria that is passed to the infant constantly during the first months of life (46, 47, 94–98). Besides being a direct source of microbes, human milk contains a group of unconjugated glycan resistant to human enzymatic digestion known as human milk oligosaccharides (HMOs). These compounds act as prebiotics for key infant gut taxonomic bacterial groups including *Bifidobacterium* (99–103) and *Bacteroides* species (104). Importantly, fermentation of HMOs results in the production of SCFAs (102, 103), increases secretory immunoglobulin A (sIgA) production, and improves gut microbiome resistance to pathogens (105, 106).

Breastfeeding also influences the training of the infant immune system through the presence of antimicrobial compounds in the human milk (lactoferrin, lysozyme) and immune effectors [sIgA, immune cells, and cytokines; (107)]. Bridgman et al. (108) demonstrated that sIgA abundance is associated with breastfeeding status in a cohort of 47 4-month-old infants. sIgA is critical for the infant gut mucosal immune defense [see Ref. (109) for a review] mainly through a process known as immune exclusion, where sIgA adheres to bacterial cells and antigens and prevents their access to the gut epithelium (110). Although this antibody is initially acquired through breastfeeding, the infant gut microbiota will ultimately stimulate its local production through the maturation of B cells (111). Notably, the risk of developing atopy is increased if B cells maturation is delayed (112–115), stressing the importance of breastfeeding in infant gut microbiome and immune development.

### Solid Food Introduction and Weaning

The introduction of solid foods constitutes the last step in early-life microbiome succession events, which leads to the consolidation of a gut microbial community that remains largely stable for the remainder of childhood and adult life. Due to the availability of new fiber sources and other substrates, transition to solid foods results in an increase of diversity and the enrichment of *Bacteroides* spp., *Clostridium* spp., *Ruminococcus* spp., *Faecalibacterium* spp., *Roseburia* spp., and *Anaerostipes* spp., as well as the reduction in *Bifidobacterium* spp. and Enterobacteriaceae (39, 40, 116, 117). Functionally, solid food introduction increases SCFA production, vitamin biosynthesis, and xenobiotic degradation (34, 39, 40). Notably, these changes coincide with important aspects of digestive development (e.g., pancreatic function and intestinal nutrient absorption) and shifts in immune development, some of which are driven by microbes. For instance, the expression of the epithelial antimicrobial granule protein, Angiogenin-4 (Ang4), and of epithelial fucosylated glycans is markedly increased during weaning in conventional but not in germ-free mice. Remarkably, colonization with *Bacteroides thetaiotaomicron*, a bacterial commensal that increases in abundance post-weaning, was able to induce both Ang4 expression and fucosylated glycan reprogramming [Ang4; (118, 119)], strongly suggesting that specific host functions have adapted to rely on microbial signals that arrive in a temporally structured manner.

Furthermore, it has been suggested that cessation of breastfeeding, rather than solid food introduction, drives the main compositional shifts that result in an “adult-like” gut microbiome. In a longitudinal study of 98 infants, an early weaning age (under 12 months) was associated with an increase in *Bacteroides* spp., *Bilophila* spp., *Roseburia* spp., *Clostridium* spp., and *Anaerostipes* spp. In comparison, breastmilk supplementation beyond this age favored a more “immature” community composition, characterized by *Bifidobacterium* spp., *Lactobacillus* spp., *Collinsella* spp., *Megasphaera* spp., and *Veillonella* spp. (39, 40).

## Infant Gut Community Diversity: An Indicator of Health?

The impact of early-life dysbiosis on the risk of developing several human diseases has led to the hypothesis that there is a critical window during which changes in the gut microbiome are most influential in immune development. During this “window of opportunity,” the infant gut harbors a highly variable and increasingly diverse microbial community of low resilience, which renders it easily disrupted by disturbances such as antibiotic treatments (41). During this period of time, a loss of diversity or change in community composition has the potential to disrupt the development of certain aspects of neonate immune system and to promote a bloom of pathogens, thus increasing the risk of developing immune-mediated and infectious diseases. However, it remains unclear if community diversity *per se* represents a robust indicator of infant gut microbiome disruption, especially since (1) there could a threshold to be crossed for the gut ecosystem to suffer a significant loss of function; and (2) diversity as a diagnostic tool provides no information on the gut microbial community composition or functional properties.

Many studies have argued that a loss of community diversity could indicate a disruption of the natural infant gut microbiome community. After birth, both the taxonomic and functional diversity of the infant bacterial microbiome have been shown to increase (88). Life-threatening diseases such as NEC have been suggested to occur as an effect of disruption of the natural succession in the infant gut microbiome after antibiotic treatment (120, 121), lowering community diversity and creating an opportunity for other bacterial groups (e.g., Gammaproteobacteria) to dominate the normal bacterial community (122, 123). At that stage, a loss of community diversity can also hinder the training of the immune system by reducing its ability to recognize commensal bacteria [see Ref. (52) for a review]. Recent studies have confirmed that a significant loss in gut microbial diversity is indicative of an increased risk of developing autoimmune diseases (80, 124). In addition, a loss of diversity can promote a long-term increase in IgE levels, which has been suggested to trigger immune-mediated disorders in mice (59).

However, it remains to be determined if the link between the development of immune diseases and the loss of microbial diversity is caused by a reduction of microbial species alone or, more precisely, by a loss of key taxonomic or functional microbial groups essential to the development of the infant immune system. The work of Arrieta et al. (13) on 319 infants in a longitudinal cohort, showed no significant relationship between fecal microbial alpha-diversity and the risk of developing asthma. Yet, four bacterial taxa (*Faecalibacterium, Lachnospira, Rothia*, and *Veillonella*), fecal acetate and deconjugated bile acids were significantly altered in babies at risk of asthma. By contrast, Kostic et al. (15) identified that a significant reduction in infant gut community alpha-diversity is a characteristic condition of the T1D state in a cohort of 33 infants predisposed to type 1 diabetes. This loss in alpha-diversity was combined with an alteration of the metabolic pathways and microbial community phylogenetic structure (15). These studies suggest that both subtle and global changes in community composition may lead to immune impairment and disease development, and that functional dysbiosis can occur independently of significant changes in community alpha-diversity.

Community alpha-diversity may also not be a reliable indicator across all human populations given its geographic variability (27). In a study comparing European to Burkina Faso children, De Filippo et al. (125) showed that the latter group had a greater gut microbial diversity and shift in community composition, potentially associated with their high fiber diet. However, other lifestyle factors and environmental exposures may also explain these differences. In addition, bacterial alpha-diversity fluctuates significantly during the first year of life, making it an unreliable ecosystem measurement unless studies are strictly age- and population matched. Further, an opposite relationship between alpha-diversity and health status occurs during the first weeks of life, where lower alpha-diversity and a predominance of a few subspecies of *Bifidobacterium longum* is associated with better growth (126).

Another factor that is rarely taken into account when assessing microbiome alpha-diversity is the impact of other non-bacterial microbes. In a unique study targeting both infant gut bacterial and fungal communities, Fujimura et al. (14) showed that infant gut bacterial alpha-diversity increased with time while the fungal alpha-diversity decreased in reciprocal correlation. This finding suggests that microbial diversity *per se* might naturally fluctuate depending on the targeted organism and that currently unexplored inter-kingdom gut microbial associations may influence these dynamics. Most interestingly, their work demonstrated that the fungal beta-diversity better predicted atopy risk than bacterial beta-diversity. Therefore, fluctuations in infant gut fungal community composition could play a role in influencing infant’s susceptibility to childhood allergies and asthma.

The increase in both taxonomic and functional diversity of the infant bacterial gut microbiome in the few months after birth appears to be associated with multiple aspects of the immune system development, providing further evidence that the immune system relies on a temporally structured succession of the gut microbiome. However, the infant gut microbial diversity *per se* might not be an indicator conveying enough information to be considered as a diagnostic tool. Notwithstanding, studies to date do suggest that the training of the immune system relies on a particular pattern of microbial diversity increasing from birth until 3 years old, and that disrupting this pattern can increase the risk of developing immune-mediated disorders. Future research disentangling the relative impact of species richness, community taxonomic, and functional composition on the retention of infant gut ecosystem emergent properties (e.g., infant immune system development) will provide key information for the development of diagnostic tools.

## Keystone Groups

In community ecology, the concept of a keystone species or group of species is described as an actor of a community that is so important to its organization and diversity that losing it provokes a massive cascade of extinctions and loss of ecosystem function (23, 24, 127). In other words, a keystone species has a remarkable impact in relation to its abundance (128). In an ecosystem, keystone species can belong to any trophic levels, from low-level species providing the resources on which a plethora of other species depends, to high-level species applying top-down regulation on the community. Keystone taxa of the infant gut microbiome contribute significantly to the ecosystem by (1) contributing to the establishment of other species; (2) by producing important metabolites including SCFAs (e.g., butyrate) that trigger local trophic cascades; (3) by improving ecosystem resistance against invading pathogenic species; and (4) by aiding in sustaining a balanced symbiosis with the host, which will in turn favor the stability of the microbial ecosystem. Because of the high inter-individual [i.e., Ref. (7)] and temporal (27, 34, 39, 40) variability of the infant gut ecosystem, identifying keystone taxa is a great challenge. Here, we discuss the potential for *Bifidobacterium* and *Bacteroides* to be keystone taxa and their role on infant immune system training.

### *Bifidobacterium* 

Bifidobacteria are dominant members of the infant gut microbiome, have a large repertoire of genes for the digestion of HMOs (104, 129), and have been isolated from maternal feces, human milk, and infant feces (130, 131), demonstrating how well adapted they are to the transmission routes and growth conditions in the infant gut. *B. longum* is the predominant species in the human gut, but several *B. longum* subspecies have different levels of adaptability and functionality in the infant gut. *B. longum* subsp*. infantis* (*Bifidobacterium infantis*), *B. longum* subsp. *longum* (*B. longum*), and *B. longum* subsp. *breve* (*B. breve*) are commonly isolated from healthy breastfed infant feces, while formula-fed infants are also colonized with *Bifidobacterium adolescentis* (132–134). Of these subspecies, *B. infantis* has the largest gene repertoire to digest all HMO structures in human milk (129). In addition, when administered as a probiotic to preterm neonates, *B. infantis* colonizes better than other subspecies (135), which may explain why clinical trials using *B. lactis* or *B. breve* as a probiotic strain in the prevention of NEC have been unsuccessful (136, 137), while 5 out of 6 trials using *B. infantis* have shown to be effective in decreasing NEC incidence in neonates (138–143).

*Bifidobacterium* species decrease the intestinal luminal pH through the production of lactate and acetate, which is considered a crucial strategy in increasing intestinal nutrient absorption (144). Acetate accounts for more than 80% of the SCFA production in the infant gut (13) [compared to over 50% in the adult gut (145)] and is a key metabolite in the early establishment of colonization resistance, by preventing infections with enteropathogens (146, 147).

Through a process known as metabolic cross-feeding, where the metabolic products of a species or group of species provide growth substrates for other populations, *Bifidobacterium’s* production of lactate and acetate sustains the growth of other species, such as *Roseburia, Eubacterium, Faecalibacterium*, and *Anaeroestipes* (148–151). In addition to this strong influence of microbe–microbe interactions, the sustained growth of other microbial species also enables the subsequent production of butyrate (152, 153). Notably, the lower abundance of colonization with *Bifidobacterium* in formula-fed babies is associated with a lower concentration of lactate and a higher gut luminal pH compared to breastfed babies (93, 154), and likely accounts for one of the root causes of the striking microbiome discrepancies observed between breastfed and formula-fed infants.

Bifidobacteria also play an exceptionally important role through its direct interactions with the developing immune system. Besides preventing enteropathogenic infections, *Bifidobacterium* species also protect the infant gut by modulating mucosal barrier function and promoting immunological and inflammatory responses (155, 156). The dominance of the infant gut microbiome by *Bifidobacterium* spp. was associated with an improved T-cell-mediated response to oral and parenteral vaccines and with lower neutrophilia at 15 weeks of age (126). *B. breve* has also evolved a mechanism to be protected from the immune system response by synthesizing a specific exopolysaccharide that increases its competitive power for space and colonization in the mouse gut (157, 158).

Collectively, *Bifidobacterium* species possess important strategies that insure their colonization at high abundance in the infant gut, prevent the growth of competing species that disfavor host fitness, and promote immune development. Due to the very high microbial inter-individual variation, and the number of subspecies found in the infant gut, it remains unclear if *Bifidobacterium* is a biomarker of infant gut health, yet the sub-species *B. infantis* may be a likely candidate.

### *Bacteroides* 

Together with *Bifidobacterium, Bacteroides* are the only groups known to use HMOs as a primary nutrient source (102, 103, 159). In addition, *Bacteroides* species are considered *generalists*—organisms with a great capacity to switch dietary nutrient sources or host-derived substrates (151). In an elegant study that followed the transcriptional profile of the human and murine symbiont, *B. thetaiotaomicron*, and the structure of murine cecal glycans, it was demonstrated that this bacterium has the gene encoding capacity to switch from digesting food sugars to foraging host mucus glycans (160). The metabolic plasticity of this species likely improves their adaptability to the fluctuating luminal conditions of the developing infant gut, especially after weaning and introduction of solid foods. Importantly, colonization with *Bacteroides* species is heavily reliant on natural events that drive succession patterns, such as vaginal birth and breastfeeding (41, 80), suggesting that *Bacteroides* spp. transmission is advantageous for both the host and members of this taxa, and that it is highly coevolved.

Certain symbionts are thought to have evolved mechanisms through which they influence the host immune system maturation in a way that is beneficial for them. An example of these mechanisms is the development of specific metabolic capacity by *B. thetaiotaomicron* (119), a microbial species previously linked with angiogenesis in the postnatal intestine development (161). This species influences the gut microbial community by regulating the epithelial glycan synthesis (162), therefore creating a specific niche for itself and for other microorganisms with similar nutrient biochemical capacity.

Another species involved in immune system development is *Bacteroides fragilis*. Its production of polysaccharide A has been shown to suppress inflammation by downregulating interleukin (IL)-17 (163). Monocolonization of germ-free mice by *B. fragilis* has been shown to balance Th1 and Th2 responses (164). In addition, these monocolonized mice showed an increase in the conversion of CD4^+^ T cells into IL-10-producing Foxp3^+^ Treg cells, which induced a strong anti-inflammatory effect during gut inflammation (165). *B. fragilis* was also demonstrated to be negatively associated with the expression of toll-like receptor-4 and with lipopolysaccharide (LPS)-induced production of multiple inflammatory cytokines and chemokines (166).

Intriguingly, recent findings on the links between *Bacteroides* and immune system training suggest that, although they are important members of the early gut microbiome, an overabundance of *Bacteroides* spp. and a corresponding increase of exposure to their LPS, result in improper stimulation of the innate immune system and in inhibition of LPS tolerance in non-obese diabetic mice. This mechanism was proposed to explain the disparity in type 1 diabetes incidence in Northern Europe, where Russian children have reduced *Bacteroides* spp. abundance and lower disease rates, compared to Finnish and Estonian children (16). This study highlights the importance of attaining a balanced stimulation of the immune response early in life and how specific gut microbes have evolved to do so in a temporally structured manner. It also underlines the complexity of disentangling the effects of particular bacterial species and higher phylogenetic groups on the emergent properties of the infant gut ecosystem and host fitness.

## Future Research

At its beginning, complexity theory suggested that ecosystems exhibiting a higher complexity were more stable when sustaining disturbances such as species loss (167, 168). However, mathematical model simulations of food webs led to the proposal that instead of focusing on the stability of individual populations within an ecosystem, a better comprehension of complex systems could be gained from studying emergent properties such as productivity, resilience, and biomass (169, 170). From this point, studies have employed multiple properties to characterize ecosystems including species richness, taxonomic composition, functional profile, the level of interactions between species of the ecosystem, and the strength of these interactions. This transition in community ecology theory mirrors the improvement of our comprehension of complex ecosystems shifting from a singular to a multi-level perspective.

In this review, we advocate that the infant gut microbiome should be considered as a complex adaptive system crucial to the maintenance of various emergent properties (e.g., infant immune system training). These ecosystem properties are hardly attributable to a single group, instead they seem to rely on a temporally structured pattern of bacterial diversity increase after birth and the succession of particular keystone groups. The properties of complex adaptive systems highlight the great challenges faced by studies of the infant gut microbiome: a system far-from equilibrium dynamics, characterized by permanent novelty and incessant adaptation, dispersed multi-level interactions, and the absence of a global controller (171). The emergent properties of this ecosystem highlight the necessity of prospective, longitudinal infant gut microbiome studies, both taxonomic and functional, which will eventually allow us to identify the critical points at which this system loses its emergent properties and reaches a state of dysbiosis, impeding adequate immune system development. In addition, there is a need to disentangle the influence of loss of taxonomic and functional diversity, as well as of shifts in keystone taxa on immune system training and subsequent disease development. From past studies, we now understand that the maturation of the immune system relies on a temporally structured dynamic, starting *in utero* with maternal effects, influenced by environmental factors (delivery mode, type of milk consumption, and solid foods) and host biology, and depending heavily on auto-correlated local interactions between microbial groups. Further understanding of this complex adaptive system will also require (1) sampling a variety of geographically distinct human populations, (2) carrying out longitudinal cohorts that sample numerous times during the first 12 months, and (3) combining amplicon-based surveys with functional assays, such as metagenomics and metabolomics.

**Figure 2:**
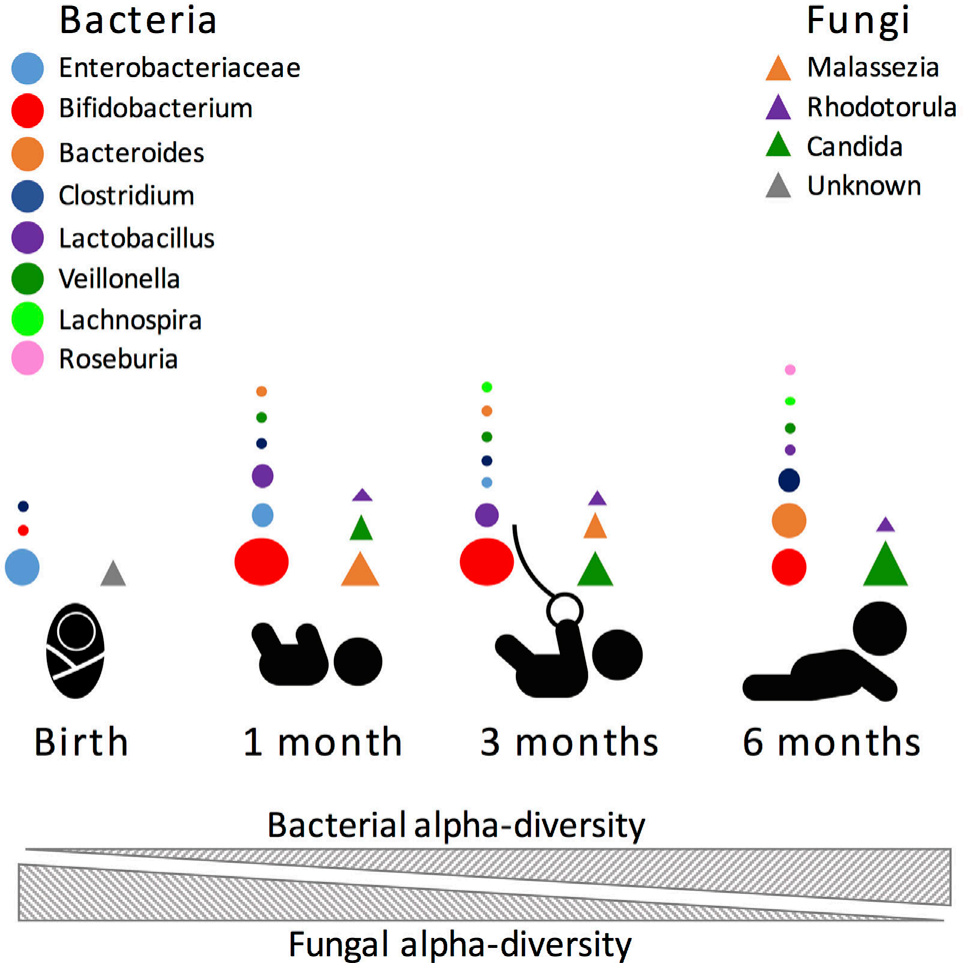
Most abundant bacterial (circles) and fungal (triangles) taxa during the first 6 months of human life. Size of the circle is proportional to the relative abundance of the bacterial taxa.

Another important influence in gut microbiome composition that remains vastly unexplored is the role of non-bacterial microorganisms. The role of the virome, the collection of viruses colonizing the host, has been previously explored in adult animals. Similarly to the bacteriome, the virome strongly interacts with the host immune system, with both positive and negative consequences for host health [see Ref. (172–174) for reviews]. However, it remains unknown what role the virome has during early-life immune development. Further, fungi, protozoans, and helminths, which are traditionally excluded from culture- and non-culture-based studies, are important and immunomodulatory members of the gut microbiome, albeit in smaller proportions than bacteria. Nonetheless, it was recently shown that fungi species are present at much higher diversity in the first months of life, compared to later months, and that this change in diversity inversely correlates with bacterial diversity [(14); Figure 2]. Future studies directed at exploring inter-kingdom gut microbial associations during early life and how these associations influence the host will provide a more global understanding of the microbial triggers influencing immune system development.

Eventually, the identification of the critical events and factors that influence microbiome resilience and function will enable the development of effective interventions aimed at maintaining and/or improving immune system development and disease prevention. Although an astounding amount of work has been carried out to understand the reliance of the immune system on the infant gut microbiome, much remains to be elucidated on the particular mechanisms responsible for this training. Improvements in our understanding will arise from continuing multidisciplinary joint efforts between immunologists, microbiologists, clinicians, bioinformaticians, and ecologists.

## Author Contributions

M-CA formulated the concept for this review, and IL-L wrote the first draft. Both authors co-wrote and revised the entire review article.

## Conflict of Interest Statement

The authors declare that this work was conducted in the absence of any commercial or financial relationships that could be construed as a potential conflict of interest.

## References

[1] Arrieta MC Stiemsma LT Amenyogbe N Brown EM Finlay B The intestinal microbiome in early life: health and disease. Front Immunol (2014) 5:427 10.3389/fimmu.2014.00427 25250028 PMC4155789

[2] Strachan DP Hay fever, hygiene, and household size. BMJ (1989) 299(6710):1259 10.1136/bmj.299.6710.1259 2513902 PMC1838109

[3] Strachan DP Family size, infection and atopy: the first decade of the ‘hygiene hypothesis’. Thorax (2000) 55(Suppl 1):S2 10.1136/thorax.55.suppl_1.S2 10943631 PMC1765943

[4] Penders J Stobberingh EE van den Brandt PA Thijs C The role of the intestinal microbiota in the development of atopic disorders. Allergy (2007) 62(11):1223–36. 10.1111/j.1398-9995.2007.01462.x 17711557

[5] Penders J Thijs C van den Brandt PA Kummeling I Snijders B Stelma F Gut microbiota composition and development of atopic manifestations in infancy: the KOALA birth cohort study. Gut (2007) 56(5):661–7. 10.1136/gut.2006.100164 17047098 PMC1942165

[6] Russell SL Finlay BB. The impact of gut microbes in allergic diseases. Curr Opin Gastroenterol (2012) 28(6):563–9. 10.1097/MOG.0b013e3283573017 23010680

[7] Hill CJ Lynch DB Murphy K Ulaszewska M Jeffery IB O’Shea CA Evolution of gut microbiota composition from birth to 24 weeks in the INFANTMET cohort. Microbiome (2017) 5(1):4. 10.1186/s40168-016-0213-y 28095889 PMC5240274

[8] Prescott SL Macaubas C Smallacombe T Holt BJ Sly PD Holt PG. Development of allergen-specific T-cell memory in atopic and normal children. Lancet (1999) 353(9148):196–200. 10.1016/S0140-6736(98)05104-6 9923875

[9] Van Der Velden V Laan M Baert M De Waal Malefyt R Neijens H Savelkoul H Selective development of a strong Th2 cytokine profile in high-risk children who develop atopy: risk factors and regulatory role of IFN-γ, IL-4 and IL-10. Clin Exp Allergy (2001) 31(7):997–1006. 10.1046/j.1365-2222.2001.01176.x 11467989

[10] Rautava S Ruuskanen O Ouwehand A Salminen S Isolauri E The hygiene hypothesis of atopic disease—an extended version. J Pediatr Gastroenterol Nutr (2004) 38(4):378–88. 10.1097/00005176-200404000-00004 15085015

[11] Costello EK Stagaman K Dethlefsen L Bohannan BJ Relman DA. The application of ecological theory toward an understanding of the human microbiome. Science (2012) 336(6086):1255–62. 10.1126/science.1224203 22674335 PMC4208626

[12] Cassir N Benamar S Khalil JB Croce O Saint-Faust M Jacquot A *Clostridium butyricum* strains and dysbiosis linked to necrotizing enterocolitis in preterm neonates. Clin Infect Dis (2015) 61(7):1107–15. 10.1093/cid/civ468 26084844

[13] Arrieta MC Stiemsma LT Dimitriu PA Thorson L Russell S Yurist-Doutsch S Early infancy microbial and metabolic alterations affect risk of childhood asthma. Sci Transl Med (2015) 7(307):307ra152. 10.1126/scitranslmed.aab2271 26424567

[14] Fujimura KE Sitarik AR Havstad S Lin DL Levan S Fadrosh D Neonatal gut microbiota associates with childhood multisensitized atopy and T cell differentiation. Nat Med (2016) 22(10):1187–91. 10.1038/nm.4176 27618652 PMC5053876

[15] Kostic AD Gevers D Siljander H Vatanen T Hyotylainen T Hamalainen AM The dynamics of the human infant gut microbiome in development and in progression toward type 1 diabetes. Cell Host Microbe (2015) 17(2):260–73. 10.1016/j.chom.2015.01.001 25662751 PMC4689191

[16] Vatanen T Kostic AD d’Hennezel E Siljander H Franzosa EA Yassour M Variation in microbiome LPS immunogenicity contributes to autoimmunity in humans. Cell (2016) 165(6):1551 10.1016/j.cell.2016.05.056 27259157

[17] Carding S Verbeke K Vipond DT Corfe BM Owen LJ. Dysbiosis of the gut microbiota in disease. Microb Ecol Health Dis (2015) 26(1):26191. 10.3402/mehd.v26.26191 25651997 PMC4315779

[18] Gevers D Kugathasan S Denson LA ázquez-Baeza YV Van Treuren W Ren B The treatment-naive microbiome in new-onset Crohn’s disease. Cell Host Microbe (2014) 15(3):382–92. 10.1016/j.chom.2014.02.005 24629344 PMC4059512

[19] Imhann F Bonder MJ Vila AV Fu J Mujagic Z Vork L Proton pump inhibitors affect the gut microbiome. Gut (2016) 65(5):740–8. 10.1136/gutjnl-2015-310376 26657899 PMC4853569

[20] Cho I Yamanishi S Cox L Methé BA Zavadil J Li K Antibiotics in early life alter the murine colonic microbiome and adiposity. Nature (2012) 488(7413):621–6. 10.1038/nature11400 22914093 PMC3553221

[21] Cox LM Yamanishi S Sohn J Alekseyenko AV Leung JM Cho I Altering the intestinal microbiota during a critical developmental window has lasting metabolic consequences. Cell (2014) 158(4):705–21. 10.1016/j.cell.2014.05.052 25126780 PMC4134513

[22] Levin SA Ecosystems and the biosphere as complex adaptive systems. Ecosystems (1998) 1(5):431–6. 10.1007/s100219900037

[23] Paine RT Food web complexity and species diversity. Am Nat (1966) 100(910):65–75. 10.1086/282400

[24] Paine RT A note on trophic complexity and community stability. Am Nat (1969) 103(929):91–3. 10.1086/282586

[25] Horn HS The ecology of secondary succession. Annu Rev Ecol Syst (1974) 5(1):25–37. 10.1146/annurev.es.05.110174.000325

[26] Spor A Koren O Ley R. Unravelling the effects of the environment and host genotype on the gut microbiome. Nat Rev Microbiol (2011) 9(4):279–90. 10.1038/nrmicro2540 21407244

[27] Yatsunenko T Rey FE Manary MJ Trehan I Dominguez-Bello MG Contreras M Human gut microbiome viewed across age and geography. Nature (2012) 486(7402):222–7. 10.1038/nature11053 22699611 PMC3376388

[28] Lozupone CA Stombaugh J Gonzalez A Ackermann G Wendel D ázquez-Baeza YV Meta-analyses of studies of the human microbiota. Genome Res (2013) 23(10):1704–14. 10.1101/gr.151803.112 23861384 PMC3787266

[29] Hooper LV Littman DR Macpherson AJ. Interactions between the microbiota and the immune system. Science (2012) 336(6086):1268–73. 10.1126/science.1223490 22674334 PMC4420145

[30] Gensollen T Iyer SS Kasper DL Blumberg RS. How colonization by microbiota in early life shapes the immune system. Science (2016) 352(6285):539–44. 10.1126/science.aad9378 27126036 PMC5050524

[31] Gray LE O’Hely M Ranganathan S Sly PD Vuillermin P. The maternal diet, gut bacteria, and bacterial metabolites during pregnancy influence offspring asthma. Front Immunol (2017) 8:365. 10.3389/fimmu.2017.00365 28408909 PMC5374203

[32] Cho I Blaser MJ. The human microbiome: at the interface of health and disease. Nat Rev Genet (2012) 13(4):260–70. 10.1038/nrg3182 22411464 PMC3418802

[33] Yu Y Lu L Sun J Petrof EO Claud EC. Preterm infant gut microbiota affects intestinal epithelial development in a humanized microbiome gnotobiotic mouse model. Am J Physiol Gastrointest Liver Physiol (2016) 311(3):G521–32. 10.1152/ajpgi.00022.2016 27492329 PMC5076002

[34] Koenig JE Spor A Scalfone N Fricker AD Stombaugh J Knight R Succession of microbial consortia in the developing infant gut microbiome. Proc Natl Acad Sci U S A (2011) 108(Suppl 1):4578–85. 10.1073/pnas.1000081107 20668239 PMC3063592

[35] Cabrera-Rubio R Collado MC Laitinen K Salminen S Isolauri E Mira A. The human milk microbiome changes over lactation and is shaped by maternal weight and mode of delivery. Am J Clin Nutr (2012) 96(3):544–51. 10.3945/ajcn.112.037382 22836031

[36] Thorburn AN McKenzie CI Shen S Stanley D Macia L Mason LJ Evidence that asthma is a developmental origin disease influenced by maternal diet and bacterial metabolites. Nat Commun (2015) 6:7320. 10.1038/ncomms8320 26102221

[37] Gomez de Aguero M Ganal-Vonarburg SC Fuhrer T Rupp S Uchimura Y Li H The maternal microbiota drives early postnatal innate immune development. Science (2016) 351(6279):1296–302. 10.1126/science.aad2571 26989247

[38] Dominguez-Bello MG Costello EK Contreras M Magris M Hidalgo G Fierer N Delivery mode shapes the acquisition and structure of the initial microbiota across multiple body habitats in newborns. Proc Natl Acad Sci U S A (2010) 107(26):11971–5. 10.1073/pnas.1002601107 20566857 PMC2900693

[39] Backhed F Roswall J Peng Y Feng Q Jia H Kovatcheva-Datchary P Dynamics and stabilization of the human gut microbiome during the first year of life. Cell Host Microbe (2015) 17(5):690–703. 10.1016/j.chom.2015.04.004 25974306

[40] Backhed F Roswall J Peng Y Feng Q Jia H Kovatcheva-Datchary P Dynamics and stabilization of the human gut microbiome during the first year of life. Cell Host Microbe (2015) 17(6):852 10.1016/j.chom.2015.05.012 26308884

[41] Yassour M Vatanen T Siljander H Hamalainen AM Harkonen T Ryhanen SJ Natural history of the infant gut microbiome and impact of antibiotic treatment on bacterial strain diversity and stability. Sci Transl Med (2016) 8(343):343ra81 10.1126/scitranslmed.aad0917 PMC5032909 27306663

[42] Palmer C Bik EM DiGiulio DB Relman DA Brown PO. Development of the human infant intestinal microbiota. PLoS Biol (2007) 5(7):e177. 10.1371/journal.pbio.0050177 17594176 PMC1896187

[43] Round JL Lee SM Li J Tran G Jabri B Chatila TA The toll-like receptor 2 pathway establishes colonization by a commensal of the human microbiota. Science (2011) 332(6032):974–7. 10.1126/science.1206095 21512004 PMC3164325

[44] Tanaka S Kobayashi T Songjinda P Tateyama A Tsubouchi M Kiyohara C Influence of antibiotic exposure in the early postnatal period on the development of intestinal microbiota. FEMS Immunol Med Microbiol (2009) 56(1):80–7. 10.1111/j.1574-695X.2009.00553.x 19385995

[45] Zeissig S Blumberg RS Life at the beginning: perturbation of the microbiota by antibiotics in early life and its role in health and disease. Nat Immunol (2014) 15(4):307–10. 10.1038/ni.2847 24646587

[46] Martín R Heilig G Zoetendal E Smidt H Rodríguez J. Diversity of the *Lactobacillus* group in breast milk and vagina of healthy women and potential role in the colonization of the infant gut. J Appl Microbiol (2007) 103(6):2638–44. 10.1111/j.1365-2672.2007.03497.x 18045446

[47] Martín R Heilig HG Zoetendal EG Jiménez E Fernández L Smidt H Cultivation-independent assessment of the bacterial diversity of breast milk among healthy women. Res Microbiol (2007) 158(1):31–7. 10.1016/j.resmic.2006.11.004 17224259

[48] Solís G de Los Reyes-Gavilan C Fernández N Margolles A Gueimonde M. Establishment and development of lactic acid bacteria and bifidobacteria microbiota in breast-milk and the infant gut. Anaerobe (2010) 16(3):307–10. 10.1016/j.anaerobe.2010.02.004 20176122

[49] Gao J Barzel B Barabási A-L Universal resilience patterns in complex networks. Nature (2016) 530(7590):307–12. 10.1038/nature16948 26887493

[50] Horner AA. Toll-like receptor ligands and atopy: a coin with at least two sides. J Allergy Clin Immunol (2006) 117(5):1133–40. 10.1016/j.jaci.2006.02.035 16675343

[51] Romagnani S Coming back to a missing immune deviation as the main explanatory mechanism for the hygiene hypothesis. J Allergy ClinImmunol (2007) 119(6):1511 10.1016/j.jaci.2007.04.005 17556059

[52] Round JL Mazmanian SK. The gut microbiota shapes intestinal immune responses during health and disease. Nat Rev Immunol (2009) 9(5):313–23. 10.1038/nri2515 19343057 PMC4095778

[53] Cummins AG Thompson FM. Effect of breast milk and weaning on epithelial growth of the small intestine in humans. Gut (2002) 51(5):748–54. 10.1136/gut.51.5.748 12377819 PMC1773445

[54] Forchielli ML Walker WA. The effect of protective nutrients on mucosal defense in the immature intestine. Acta Paediatr Suppl (2005) 94(449):74–83. 10.1080/08035320510043592 16214770

[55] Lyons A O’mahony D O’brien F MacSharry J Sheil B Ceddia M Bacterial strain-specific induction of Foxp3+ T regulatory cells is protective in murine allergy models. Clin Exp Allergy (2010) 40(5):811–9. 10.1111/j.1365-2222.2009.03437.x 20067483

[56] Turroni F Peano C Pass DA Foroni E Severgnini M Claesson MJ Diversity of bifidobacteria within the infant gut microbiota. PLoS One (2012) 7(5):e36957 10.1371/journal.pone.0036957 22606315 PMC3350489

[57] Isolauri E Sutas Y Kankaanpaa P Arvilommi H Salminen S Probiotics: effects on immunity. Am J Clin Nutr (2001) 73(2 Suppl):444S–50S. 11157355 10.1093/ajcn/73.2.444s

[58] Arnold IC Dehzad N Reuter S Martin H Becher B Taube C *Helicobacter pylori* infection prevents allergic asthma in mouse models through the induction of regulatory T cells. J Clin Invest (2011) 121(8):3088–93. 10.1172/JCI45041 21737881 PMC3148731

[59] Cahenzli J Koller Y Wyss M Geuking MB McCoy KD. Intestinal microbial diversity during early-life colonization shapes long-term IgE levels. Cell Host Microbe (2013) 14(5):559–70. 10.1016/j.chom.2013.10.004 24237701 PMC4049278

[60] Russell SL Gold MJ Hartmann M Willing BP Thorson L Wlodarska M Early life antibiotic-driven changes in microbiota enhance susceptibility to allergic asthma. EMBO Rep (2012) 13(5):440–7. 10.1038/embor.2012.32 22422004 PMC3343350

[61] Livanos AE Greiner TU Vangay P Pathmasiri W Stewart D McRitchie S Antibiotic-mediated gut microbiome perturbation accelerates development of type 1 diabetes in mice. Nat Microbiol (2016) 1(11):16140. 10.1038/nmicrobiol.2016.140 27782139 PMC5808443

[62] Hill DA Siracusa MC Abt MC Kim BS Kobuley D Kubo M Commensal bacteria-derived signals regulate basophil hematopoiesis and allergic inflammation. Nat Med (2012) 18(4):538–46. 10.1038/nm.2657 22447074 PMC3321082

[63] Russell SL Gold MJ Reynolds LA Willing BP Dimitriu P Thorson L Perinatal antibiotic-induced shifts in gut microbiota have differential effects on inflammatory lung diseases. J Allergy Clin Immunol (2015) 135(1):100–9. 10.1016/j.jaci.2014.06.027 25145536

[64] Li J Yang K Ju T Ho T McKay CA Gao Y Early life antibiotic exposure affects pancreatic islet development and metabolic regulation. Sci Rep (2017) 7:41778 10.1038/srep41778 28150721 PMC5288777

[65] Scheer S Medina TS Murison A Taves MD Antignano F Chenery A Early-life antibiotic treatment enhances the pathogenicity of CD4+ T cells during intestinal inflammation. J Leukoc Biol (2017) 101(4):893–900. 10.1189/jlb.3MA0716-334RR 28034915

[66] Corrêa-Oliveira R Fachi JL Vieira A Sato FT Vinolo MAR Regulation of immune cell function by short-chain fatty acids. Clin Transl Immunol (2016) 5(4):e73 10.1038/cti.2016.17 PMC4855267 27195116

[67] Nicholson JK Holmes E Kinross J Burcelin R Gibson G Jia W Host-gut microbiota metabolic interactions. Science (2012) 336(6086):1262–7. 10.1126/science.1223813 22674330

[68] Smith PM Howitt MR Panikov N Michaud M Gallini CA Bohlooly-y M The microbial metabolites, short-chain fatty acids, regulate colonic Treg cell homeostasis. Science (2013) 341(6145):569–73. 10.1126/science.1241165 23828891 PMC3807819

[69] Arpaia N Campbell C Fan X Dikiy S van der Veeken J deRoos P Metabolites produced by commensal bacteria promote peripheral regulatory T-cell generation. Nature (2013) 504(7480):451–5. 10.1038/nature12726 24226773 PMC3869884

[70] Furusawa Y Obata Y Fukuda S Endo TA Nakato G Takahashi D Commensal microbe-derived butyrate induces the differentiation of colonic regulatory T cells. Nature (2013) 504(7480):446–50. 10.1038/nature12721 24226770

[71] Kim YG Sakamoto K Seo SU Pickard JM Gillilland MG III Pudlo NA Neonatal acquisition of *Clostridia* species protects against colonization by bacterial pathogens. Science (2017) 356(6335):315–9. 10.1126/science.aag2029 28428425 PMC6082366

[72] Bomba L Minuti A Moisá SJ Trevisi E Eufemi E Lizier M Gut response induced by weaning in piglet features marked changes in immune and inflammatory response. Funct Integr Genomics (2014) 14(4):657–71. 10.1007/s10142-014-0396-x 25199657

[73] Parigi SM Eldh M Larssen P Gabrielsson S Villablanca EJ. Breast milk and solid food shaping intestinal immunity. Front Immunol (2015) 6:415. 10.3389/fimmu.2015.00415 26347740 PMC4541369

[74] Clements FE Plant Succession: An Analysis of the Development of Vegetation. Washington: Carnegie Institution of Washington (1916).

[75] Aagaard K Ma J Antony KM Ganu R Petrosino J Versalovic J. The placenta harbors a unique microbiome. Sci Transl Med (2014) 6(237):237ra65. 10.1126/scitranslmed.3008599 24848255 PMC4929217

[76] Collado MC Rautava S Aakko J Isolauri E Salminen S. Human gut colonisation may be initiated in utero by distinct microbial communities in the placenta and amniotic fluid. Sci Rep (2016) 6:23129. 10.1038/srep23129 27001291 PMC4802384

[77] Perez-Muñoz ME Arrieta M-C Ramer-Tait AE Walter J A critical assessment of the sterile womb and in utero colonization hypotheses: implications for research on the pioneer infant microbiome. Microbiome (2017) 5(1):48 10.1186/s40168-017-0268-4 28454555 PMC5410102

[78] Dominguez-Bello MG Cienfuentes C Romero R Garcia P Gomez I Mago V PCR detection of *Helicobacter pylori* in string-absorbed gastric juice. FEMS Microbiol Lett (2001) 198(1):15–6. 10.1111/j.1574-6968.2001.tb10612.x 11325547

[79] Bezirtzoglou E. The intestinal microflora during the first weeks of life. Anaerobe (1997) 3(2–3):173–7. 10.1006/anae.1997.0102 16887585

[80] Jakobsson HE Abrahamsson TR Jenmalm MC Harris K Quince C Jernberg C Decreased gut microbiota diversity, delayed Bacteroidetes colonisation and reduced Th1 responses in infants delivered by caesarean section. Gut (2014) 63(4):559–66. 10.1136/gutjnl-2012-303249 23926244

[81] Chen T Long W Zhang C Liu S Zhao L Hamaker B Fiber utilizing capacity varies with *Prevotella* versus *Bacteroides* enterotypes. FASEB J (2016) 30(1 Suppl):683.2. 10.1038/s41598-017-02995-4 PMC5453967 28572676

[82] Salminen S Gibson G McCartney A Isolauri E Influence of mode of delivery on gut microbiota composition in seven year old children. Gut (2004) 53(9):1388–9. 10.1136/gut.2004.041640 PMC1774211 15306608

[83] Adlerberth I Lindberg E Åberg N Hesselmar B Saalman R Strannegård I-L Reduced enterobacterial and increased staphylococcal colonization of the infantile bowel: an effect of hygienic lifestyle? Pediatr Res (2006) 59(1):96–101. 10.1203/01.pdr.0000191137.12774.b2 16380405

[84] Penders J Thijs C Vink C Stelma FF Snijders B Kummeling I Factors influencing the composition of the intestinal microbiota in early infancy. Pediatrics (2006) 118(2):511–21. 10.1542/peds.2005-2824 16882802

[85] Biasucci G Benenati B Morelli L Bessi E Boehm G Cesarean delivery may affect the early biodiversity of intestinal bacteria. J Nutr (2008) 138(9):1796S–800S. 18716189 10.1093/jn/138.9.1796S

[86] Matamoros S Gras-Leguen C Le Vacon F Potel G de La Cochetiere M-F Development of intestinal microbiota in infants and its impact on health. Trends Microbiol (2013) 21(4):167–73. 10.1016/j.tim.2012.12.001 23332725

[87] Bokulich NA Chung J Battaglia T Henderson N Jay M Li H Antibiotics, birth mode, and diet shape microbiome maturation during early life. Sci Transl Med (2016) 8(343):343ra82. 10.1126/scitranslmed.aad7121 27306664 PMC5308924

[88] Chu DM Ma J Prince AL Antony KM Seferovic MD Aagaard KM. Maturation of the infant microbiome community structure and function across multiple body sites and in relation to mode of delivery. Nat Med (2017) 23(3):314–26. 10.1038/nm.4272 28112736 PMC5345907

[89] La Rosa PS Warner BB Zhou Y Weinstock GM Sodergren E Hall-Moore CM Patterned progression of bacterial populations in the premature infant gut. Proc Natl Acad Sci U S A (2014) 111(34):12522–7. 10.1073/pnas.1409497111 25114261 PMC4151715

[90] Penders J Vink C Driessen C London N Thijs C Stobberingh EE. Quantification of *Bifidobacterium* spp., *Escherichia coli* and *Clostridium* difficile in faecal samples of breast-fed and formula-fed infants by real-time PCR. FEMS Microbiol Lett (2005) 243(1):141–7. 10.1016/j.femsle.2004.11.052 15668012

[91] Adlerberth I Wold AE. Establishment of the gut microbiota in Western infants. Acta Paediatr (2009) 98(2):229–38. 10.1111/j.1651-2227.2008.01060.x 19143664

[92] Fallani M Young D Scott J Norin E Amarri S Adam R Intestinal microbiota of 6-week-old infants across Europe: geographic influence beyond delivery mode, breast-feeding, and antibiotics. J Pediatr Gastroenterol Nutr (2010) 51(1):77–84. 10.1097/MPG.0b013e3181d1b11e 20479681

[93] Bezirtzoglou E Tsiotsias A Welling GW. Microbiota profile in feces of breast- and formula-fed newborns by using fluorescence in situ hybridization (FISH). Anaerobe (2011) 17(6):478–82. 10.1016/j.anaerobe.2011.03.009 21497661

[94] Heikkilä M Saris P. Inhibition of *Staphylococcus aureus* by the commensal bacteria of human milk. J Appl Microbiol (2003) 95(3):471–8. 10.1046/j.1365-2672.2003.02002.x 12911694

[95] Gueimonde M Laitinen K Salminen S Isolauri E. Breast milk: a source of bifidobacteria for infant gut development and maturation? Neonatology (2007) 92(1):64–6. 10.1159/000100088 17596738

[96] Collado M Delgado S Maldonado A Rodríguez J Assessment of the bacterial diversity of breast milk of healthy women by quantitative real-time PCR. Lett Appl Microbiol (2009) 48(5):523–8. 10.1111/j.1472-765X.2009.02567.x 19228290

[97] Martín R Jiménez E Heilig H Fernández L Marín ML Zoetendal EG Isolation of bifidobacteria from breast milk and assessment of the bifidobacterial population by PCR-denaturing gradient gel electrophoresis and quantitative real-time PCR. Appl Environ Microbiol (2009) 75(4):965–9. 10.1128/AEM.02063-08 19088308 PMC2643565

[98] Hunt KM Foster JA Forney LJ Schütte UM Beck DL Abdo Z Characterization of the diversity and temporal stability of bacterial communities in human milk. PLoS One (2011) 6(6):e21313. 10.1371/journal.pone.0021313 21695057 PMC3117882

[99] Ward RE Niaeonuevo M Mills DA Lebrilla CB German JB Research article in vitro fermentability of human milk oligosaccharides by several strains of bifidobacteria. Mol Nutr Food Res (2007) 51:1398–405. 10.1002/mnfr.200700150 17966141

[100] Sela DA Mills DA. Nursing our microbiota: molecular linkages between bifidobacteria and milk oligosaccharides. Trends Microbiol (2010) 18(7):298–307. 10.1016/j.tim.2010.03.008 20409714 PMC2902656

[101] Barile D Rastall RA. Human milk and related oligosaccharides as prebiotics. Curr Opin Biotechnol (2013) 24(2):214–9. 10.1016/j.copbio.2013.01.008 23434179

[102] Yu Z-T Chen C Kling DE Liu B McCoy JM Merighi M The principal fucosylated oligosaccharides of human milk exhibit prebiotic properties on cultured infant microbiota. Glycobiology (2013) 23(2):169–77. 10.1093/glycob/cws138 23028202 PMC3531294

[103] Yu ZT Chen C Newburg DS. Utilization of major fucosylated and sialylated human milk oligosaccharides by isolated human gut microbes. Glycobiology (2013) 23(11):1281–92. 10.1093/glycob/cwt065 24013960 PMC3796377

[104] Marcobal A Sonnenburg JL. Human milk oligosaccharide consumption by intestinal microbiota. Clin Microbiol Infect (2012) 18(Suppl 4):12–5. 10.1111/j.1469-0691.2012.03863.x 22647041 PMC3671919

[105] Newburg DS Walker WA. Protection of the neonate by the innate immune system of developing gut and of human milk. Pediatr Res (2007) 61(1):2–8. 10.1203/01.pdr.0000250274.68571.18 17211132

[106] Newburg DS. Neonatal protection by an innate immune system of human milk consisting of oligosaccharides and glycans. J Anim Sci (2009) 87(13 Suppl):26–34. 10.2527/jas.2008-1347 19028867

[107] Moles L Gomez M Jimenez E Fernandez L Bustos G Chaves F Preterm infant gut colonization in the neonatal ICU and complete restoration 2 years later. Clin Microbiol Infect (2015) 21(10):936.e1–10. 10.1016/j.cmi.2015.06.003 26086569

[108] Bridgman SL Konya T Azad MB Sears MR Becker AB Turvey SE Infant gut immunity: a preliminary study of IgA associations with breastfeeding. J Dev Orig Health Dis (2016) 7(1):68–72. 10.1017/S2040174415007862 26690933

[109] Brandtzaeg P. Secretory IgA: designed for anti-microbial defense. Front Immunol (2013) 4:222. 10.3389/fimmu.2013.00222 23964273 PMC3734371

[110] Brandtzaeg P Mucosal immunity: induction, dissemination, and effector functions. Scand J Immunol (2009) 70(6):505–15. 10.1111/j.1365-3083.2009.02319.x 19906191

[111] Macpherson AJ Uhr T. Induction of protective IgA by intestinal dendritic cells carrying commensal bacteria. Science (2004) 303(5664):1662–5. 10.1126/science.1091334 15016999

[112] Kukkonen K Kuitunen M Haahtela T Korpela R Poussa T Savilahti E High intestinal IgA associates with reduced risk of IgE-associated allergic diseases. Pediatr Allergy Immunol (2010) 21(1 Pt I):67–73. 10.1111/j.1399-3038.2009.00907.x 19566584

[113] Sandin A Björkstén B Böttcher MF Englund E Jenmalm MC Bråbäck L High salivary secretory IgA antibody levels are associated with less late-onset wheezing in IgE-sensitized infants. Pediatr Allergy Immunol (2011) 22(5):477–81. 10.1111/j.1399-3038.2010.01106.x 21332801

[114] Orivuori L Loss G Roduit C Dalphin JC Depner M Genuneit J Soluble immunoglobulin A in breast milk is inversely associated with atopic dermatitis at early age: the PASTURE cohort study. Clin Exp Allergy (2014) 44(1):102–12. 10.1111/cea.12199 24102779

[115] Orivuori L Mustonen K de Goffau MC Hakala S Paasela M Roduit C High level of fecal calprotectin at age 2 months as a marker of intestinal inflammation predicts atopic dermatitis and asthma by age 6. Clin Exp Allergy (2015) 45(5):928–39. 10.1111/cea.12522 25758537

[116] Fallani M Amarri S Uusijarvi A Adam R Khanna S Aguilera M Determinants of the human infant intestinal microbiota after the introduction of first complementary foods in infant samples from five European centres. Microbiology (2011) 157(5):1385–92. 10.1099/mic.0.042143-0 21330436

[117] Vallès Y Artacho A Pascual-García A Ferrús ML Gosalbes MJ Abellán JJ Microbial succession in the gut: directional trends of taxonomic and functional change in a birth cohort of Spanish infants. PLoS Genet (2014) 10(6):e1004406. 10.1371/journal.pgen.1004406 24901968 PMC4046925

[118] Bry L Falk PG Midtvedt T Gordon JI. A model of host-microbial interactions in an open mammalian ecosystem. Science (1996) 273(5280):1380. 10.1126/science.273.5280.1380 8703071

[119] Hooper LV Stappenbeck TS Hong CV Gordon JI. Angiogenins: a new class of microbicidal proteins involved in innate immunity. Nat Immunol (2003) 4(3):269–73. 10.1038/ni888 12548285

[120] Cotten CM Taylor S Stoll B Goldberg RN Hansen NI Sánchez PJ Prolonged duration of initial empirical antibiotic treatment is associated with increased rates of necrotizing enterocolitis and death for extremely low birth weight infants. Pediatrics (2009) 123(1):58–66. 10.1542/peds.2007-3423 19117861 PMC2760222

[121] Alexander VN Northrup V Bizzarro MJ. Antibiotic exposure in the newborn intensive care unit and the risk of necrotizing enterocolitis. J Pediatr (2011) 159(3):392–7. 10.1016/j.jpeds.2011.02.035 21489560 PMC3137655

[122] Wang Y Hoenig JD Malin KJ Qamar S Petrof EO Sun J 16S rRNA gene-based analysis of fecal microbiota from preterm infants with and without necrotizing enterocolitis. ISME J (2009) 3(8):944–54. 10.1038/ismej.2009.37 19369970 PMC2713796

[123] Mai V Young CM Ukhanova M Wang X Sun Y Casella G Fecal microbiota in premature infants prior to necrotizing enterocolitis. PLoS One (2011) 6(6):e20647. 10.1371/journal.pone.0020647 21674011 PMC3108958

[124] Abrahamsson TR Jakobsson HE Andersson AF Bjorksten B Engstrand L Jenmalm MC. Low gut microbiota diversity in early infancy precedes asthma at school age. Clin Exp Allergy (2014) 44(6):842–50. 10.1111/cea.12253 24330256

[125] De Filippo C Cavalieri D Di Paola M Ramazzotti M Poullet JB Massart S Impact of diet in shaping gut microbiota revealed by a comparative study in children from Europe and rural Africa. Proc Natl Acad Sci U S A (2010) 107(33):14691–6. 10.1073/pnas.1005963107 20679230 PMC2930426

[126] Huda MN Lewis Z Kalanetra KM Rashid M Ahmad SM Raqib R Stool microbiota and vaccine responses of infants. Pediatrics (2014) 134(2):e362–72. 10.1542/peds.2013-3937 25002669 PMC4187229

[127] Mills LS Soulé ME Doak DF The keystone-species concept in ecology and conservation. Bioscience (1993) 43(4):219–24. 10.2307/1312122

[128] Power ME Tilman D Estes JA Menge BA Bond WJ Mills LS Challenges in the quest for keystones. Bioscience (1996) 46(8):609–20. 10.2307/1312990

[129] Underwood MA German JB Lebrilla CB Mills DA. *Bifidobacterium longum* subspecies infantis: champion colonizer of the infant gut. Pediatr Res (2015) 77(1–2):229–35. 10.1038/pr.2014.156 25303277 PMC4350908

[130] Jost T Lacroix C Braegger C Chassard C. Assessment of bacterial diversity in breast milk using culture-dependent and culture-independent approaches. Br J Nutr (2013) 110(7):1253–62. 10.1017/S0007114513000597 23507238

[131] Jost T Lacroix C Braegger C Chassard C. Stability of the maternal gut microbiota during late pregnancy and early lactation. Curr Microbiol (2014) 68(4):419–27. 10.1007/s00284-013-0491-6 24258611

[132] Sakata S Tonooka T Ishizeki S Takada M Sakamoto M Fukuyama M Culture-independent analysis of fecal microbiota in infants, with special reference to *Bifidobacterium* species. FEMS Microbiol Lett (2005) 243(2):417–23. 10.1016/j.femsle.2005.01.002 15686844

[133] Turroni F Foroni E Pizzetti P Giubellini V Ribbera A Merusi P Exploring the diversity of the bifidobacterial population in the human intestinal tract. Appl Environ Microbiol (2009) 75(6):1534–45. 10.1128/AEM.02216-08 19168652 PMC2655441

[134] Roger LC Costabile A Holland DT Hoyles L McCartney AL Examination of faecal *Bifidobacterium* populations in breast- and formula-fed infants during the first 18 months of life. Microbiology (2010) 156(Pt 11):3329–41. 10.1099/mic.0.043224-0 20864478

[135] Underwood MA Kalanetra KM Bokulich NA Lewis ZT Mirmiran M Tancredi DJ A comparison of two probiotic strains of bifidobacteria in premature infants. J Pediatr (2013) 163(6):1585–1591.e9. 10.1016/j.jpeds.2013.07.017 23993139 PMC3842430

[136] Szajewska H Guandalini S Morelli L Van Goudoever JB Walker A. Effect of *Bifidobacterium animalis* subsp *lactis* supplementation in preterm infants: a systematic review of randomized controlled trials. J Pediatr Gastroenterol Nutr (2010) 51(2):203–9. 10.1097/MPG.0b013e3181dc0d93 20543719 PMC4507410

[137] Costeloe K Hardy P Juszczak E Wilks M Millar MR Probiotics in Preterm Infants Study Collaborative Group. *Bifidobacterium breve* BBG-001 in very preterm infants: a randomised controlled phase 3 trial. Lancet (2016) 387(10019):649–60. 10.1016/S0140-6736(15)01027-2 26628328

[138] Bin-Nun A Bromiker R Wilschanski M Kaplan M Rudensky B Caplan M Oral probiotics prevent necrotizing enterocolitis in very low birth weight neonates. J Pediatr (2005) 147(2):192–6. 10.1016/j.jpeds.2005.03.054 16126048

[139] Lin HC Su BH Chen AC Lin TW Tsai CH Yeh TF Oral probiotics reduce the incidence and severity of necrotizing enterocolitis in very low birth weight infants. Pediatrics (2005) 115(1):1–4. 10.1542/peds.2004-1463 15629973

[140] Samanta M Sarkar M Ghosh P Ghosh J Sinha M Chatterjee S. Prophylactic probiotics for prevention of necrotizing enterocolitis in very low birth weight newborns. J Trop Pediatr (2009) 55(2):128–31. 10.1093/tropej/fmn091 18842610

[141] Underwood MA Salzman NH Bennett SH Barman M Mills DA Marcobal A A randomized placebo-controlled comparison of 2 prebiotic/probiotic combinations in preterm infants: impact on weight gain, intestinal microbiota, and fecal short-chain fatty acids. J Pediatr Gastroenterol Nutr (2009) 48(2):216–25. 10.1097/MPG.0b013e31818de195 19179885 PMC2743418

[142] Fernandez-Carrocera LA Solis-Herrera A Cabanillas-Ayon M Gallardo-Sarmiento RB Garcia-Perez CS Montano-Rodriguez R Double-blind, randomised clinical assay to evaluate the efficacy of probiotics in preterm newborns weighing less than 1500 g in the prevention of necrotising enterocolitis. Arch Dis Child Fetal Neonatal Ed (2013) 98(1):F5–9. 10.1136/archdischild-2011-300435 22556209

[143] Jacobs SE Tobin JM Opie GF Donath S Tabrizi SN Pirotta M Probiotic effects on late-onset sepsis in very preterm infants: a randomized controlled trial. Pediatrics (2013) 132(6):1055–62. 10.1542/peds.2013-1339 24249817

[144] Macfarlane GT Macfarlane S. Bacteria, colonic fermentation, and gastrointestinal health. J AOAC Int (2012) 95(1):50–60. 10.5740/jaoacint.SGE_Macfarlane 22468341

[145] Louis P Scott KP Duncan SH Flint HJ. Understanding the effects of diet on bacterial metabolism in the large intestine. J Appl Microbiol (2007) 102(5):1197–208. 10.1111/j.1365-2672.2007.03322.x 17448155

[146] Fukuda S Toh H Hase K Oshima K Nakanishi Y Yoshimura K Bifidobacteria can protect from enteropathogenic infection through production of acetate. Nature (2011) 469(7331):543–7. 10.1038/nature09646 21270894

[147] Fukuda S Toh H Taylor TD Ohno H Hattori M. Acetate-producing bifidobacteria protect the host from enteropathogenic infection via carbohydrate transporters. Gut Microbes (2012) 3(5):449–54. 10.4161/gmic.21214 22825494

[148] Duncan SH Louis P Flint HJ. Lactate-utilizing bacteria, isolated from human feces, that produce butyrate as a major fermentation product. Appl Environ Microbiol (2004) 70(10):5810–7. 10.1128/AEM.70.10.5810-5817.2004 15466518 PMC522113

[149] Belenguer A Duncan SH Calder AG Holtrop G Louis P Lobley GE Two routes of metabolic cross-feeding between *Bifidobacterium* adolescentis and butyrate-producing anaerobes from the human gut. Appl Environ Microbiol (2006) 72(5):3593–9. 10.1128/AEM.72.5.3593-3599.2006 16672507 PMC1472403

[150] Falony G Vlachou A Verbrugghe K De Vuyst L. Cross-feeding between *Bifidobacterium longum* BB536 and acetate-converting, butyrate-producing colon bacteria during growth on oligofructose. Appl Environ Microbiol (2006) 72(12):7835–41. 10.1128/AEM.01296-06 17056678 PMC1694233

[151] Flint HJ Duncan SH Scott KP Louis P. Links between diet, gut microbiota composition and gut metabolism. Proc Nutr Soc (2015) 74(1):13–22. 10.1017/S0029665114001463 25268552

[152] Duncan SH Barcenilla A Stewart CS Pryde SE Flint HJ. Acetate utilization and butyryl coenzyme A (CoA):acetate-CoA transferase in butyrate-producing bacteria from the human large intestine. Appl Environ Microbiol (2002) 68(10):5186–90. 10.1128/AEM.68.10.5186-5190.2002 12324374 PMC126392

[153] Louis P Hold GL Flint HJ. The gut microbiota, bacterial metabolites and colorectal cancer. Nat Rev Microbiol (2014) 12(10):661–72. 10.1038/nrmicro3344 25198138

[154] Ogawa K Ben RA Pons S de Paolo MI Bustos Fernandez L. Volatile fatty acids, lactic acid, and pH in the stools of breast-fed and bottle-fed infants. J Pediatr Gastroenterol Nutr (1992) 15(3):248–52. 10.1097/00005176-199210000-00004 1432461

[155] Butel M-J Suau A Campeotto F Magne F Aires J Ferraris L Conditions of bifidobacterial colonization in preterm infants: a prospective analysis. J Pediatr Gastroenterol Nutr (2007) 44(5):577–82. 10.1097/MPG.0b013e3180406b20 17460489

[156] Abdulkadir B Nelson A Skeath T Marrs EC Perry JD Cummings SP Routine use of probiotics in preterm infants: longitudinal impact on the microbiome and metabolome. Neonatology (2016) 109(4):239–47. 10.1159/000442936 26859305

[157] Fanning S Hall LJ Cronin M Zomer A MacSharry J Goulding D Bifidobacterial surface-exopolysaccharide facilitates commensal-host interaction through immune modulation and pathogen protection. Proc Natl Acad Sci U S A (2012) 109(6):2108–13. 10.1073/pnas.1115621109 22308390 PMC3277520

[158] Fanning S Hall LJ van Sinderen D. *Bifidobacterium breve* UCC2003 surface exopolysaccharide production is a beneficial trait mediating commensal-host interaction through immune modulation and pathogen protection. Gut Microbes (2012) 3(5):420–5. 10.4161/gmic.20630 22713271

[159] Marcobal A Barboza M Sonnenburg ED Pudlo N Martens EC Desai P *Bacteroides* in the infant gut consume milk oligosaccharides via mucus-utilization pathways. Cell Host Microbe (2011) 10(5):507–14. 10.1016/j.chom.2011.10.007 22036470 PMC3227561

[160] Sonnenburg JL Xu J Leip DD Chen CH Westover BP Weatherford J Glycan foraging in vivo by an intestine-adapted bacterial symbiont. Science (2005) 307(5717):1955–9. 10.1126/science.1109051 15790854

[161] Stappenbeck TS Hooper LV Gordon JI. Developmental regulation of intestinal angiogenesis by indigenous microbes via Paneth cells. Proc Natl Acad Sci U S A (2002) 99(24):15451–5. 10.1073/pnas.202604299 12432102 PMC137737

[162] Hooper LV Gordon JI. Commensal host-bacterial relationships in the gut. Science (2001) 292(5519):1115–8. 10.1126/science.1058709 11352068

[163] Round JL Mazmanian SK. Inducible Foxp3+ regulatory T-cell development by a commensal bacterium of the intestinal microbiota. Proc Natl Acad Sci U S A (2010) 107(27):12204–9. 10.1073/pnas.0909122107 20566854 PMC2901479

[164] Mazmanian SK Liu CH Tzianabos AO Kasper DL. An immunomodulatory molecule of symbiotic bacteria directs maturation of the host immune system. Cell (2005) 122(1):107–18. 10.1016/j.cell.2005.05.007 16009137

[165] Telesford KM Yan W Ochoa-Reparaz J Pant A Kircher C Christy MA A commensal symbiotic factor derived from *Bacteroides fragilis* promotes human CD39+ Foxp3+ T cells and Treg function. Gut Microbes (2015) 6(4):234–42. 10.1080/19490976.2015.1056973 26230152 PMC4615798

[166] Sjogren YM Tomicic S Lundberg A Bottcher MF Bjorksten B verremark-Ekstrom ES Influence of early gut microbiota on the maturation of childhood mucosal and systemic immune responses. Clin Exp Allergy (2009) 39(12):1842–51. 10.1111/j.1365-2222.2009.03326.x 19735274

[167] MacArthur R Fluctuations of animal populations and a measure of community stability. Ecology (1955) 36(3):533–6.

[168] Elton CC The reasons for conservation. The Ecology of Invasions by Animals and Plants. Netherlands, London, UK: Springer; Methuen and Co. (1958). p. 143–53. 10.1007/978-94-009-5851-7

[169] May RM Stability and Complexity in Model Ecosystems. Princeton: Princeton University Press (1973).

[170] Tilman D The ecological consequences of changes in biodiversity: a search for general principles. Ecology (1999) 80(5):1455–74. 10.2307/176540

[171] Arthur WB Durlauf SN Lane DA The Economy As an Evolving Complex System II. Reading, MA: Addison-Wesley (1997).

[172] Kernbauer E Cadwell K. Autophagy, viruses, and intestinal immunity. Curr Opin Gastroenterol (2014) 30(6):539–46. 10.1097/MOG.0000000000000121 25291356 PMC4211104

[173] Virgin HW The virome in mammalian physiology and disease. Cell (2014) 157(1):142–50. 10.1016/j.cell.2014.02.032 24679532 PMC3977141

[174] Cadwell K The virome in host health and disease. Immunity (2015) 42(5):805–13. 10.1016/j.immuni.2015.05.003 25992857 PMC4578625

